# Crystal structure of methyl (2*Z*)-2-{[*N*-(2-formyl­phen­yl)-4-methyl­benzene­sulfonamido]­meth­yl}-3-(4-meth­oxy­phen­yl)prop-2-enoate

**DOI:** 10.1107/S2056989015024172

**Published:** 2015-12-31

**Authors:** Ankur Trigunait, Kannan Damodharan, Bakthadoss Manickam, Gunasekaran Krishnasamy

**Affiliations:** a Centre of Advanced Study in Crystallography and Biophysics, University of Madras, Guindy Campus, Chennai 600 025, India; b Department of Organic Chemistry, University of Madras, Guindy Campus, Chennai 600 025, India

**Keywords:** crystal structure, sulfonamide, Thorpe–Ingold effect

## Abstract

In the title compound, C_26_H_25_NO_6_S, the S atom shows a distorted tetra­hedral geometry, with O—S—O [119.46 (9)°] and N—S—C [107.16 (7)°] angles deviating from ideal tetra­hedral values, a fact attributed to the Thorpe–Ingold effect. The sulfonyl-bound phenyl ring forms dihedral angles of 41.1 (1) and 83.3 (1)°, respectively, with the formyl­phenyl and phenyl rings. The dihedral angle between formyl­phenyl and phenyl rings is 47.6 (1)°. The crystal packing features C—H⋯O hydrogen-bond inter­actions.

## Related literature   

For background to the pharmacological uses of sulfonamides, see: Korolkovas *et al.* (1988 ▸); Mandell & Sande (1992 ▸). For the anti­filarial activity of sulfonamide derivatives, see: Radembino *et al.* (1997 ▸); For related structures, see: Ranjith *et al.* (2009 ▸); Madhanraj *et al.* (2011 ▸). For the Thorpe–Ingold effect, see: Bassindale *et al.* (1984 ▸).
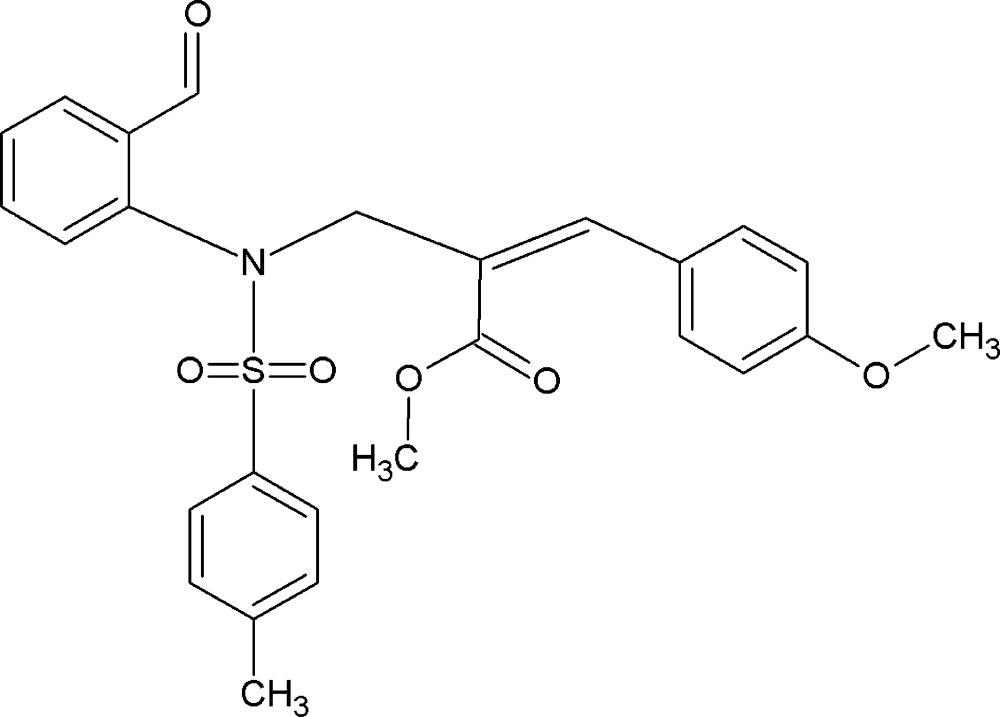


## Experimental   

### Crystal data  

C_26_H_25_NO_6_S
*M* *_r_* = 479.53
Triclinic, 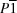

*a* = 8.3501 (2) Å
*b* = 8.4859 (2) Å
*c* = 17.6814 (4) Å
α = 84.424 (1)°
β = 80.952 (1)°
γ = 80.954 (1)°
*V* = 1218.52 (5) Å^3^
*Z* = 2
Mo *K*α radiation
μ = 0.17 mm^−1^
*T* = 293 K
0.25 × 0.20 × 0.20 mm

### Data collection  

Bruker Kappa APEXII CCD diffractometer
Absorption correction: multi-scan (*SADABS*; Bruker 2004 ▸) *T*_min_ = 0.979, *T*_max_ = 0.983
23816 measured reflections
5519 independent reflections
4232 reflections with *I* > 2σ(*I*)
*R*_int_ = 0.028

### Refinement  

*R*[*F* ^2^ > 2σ(*F* ^2^)] = 0.044
*wR*(*F* ^2^) = 0.135
*S* = 1.02
5519 reflections
314 parameters
13 restraints
H-atom parameters constrained
Δρ_max_ = 0.34 e Å^−3^
Δρ_min_ = −0.36 e Å^−3^

### 

Data collection: *APEX2* (Bruker, 2004 ▸); cell refinement: *APEX2* and *SAINT* (Bruker, 2004 ▸); data reduction: *SAINT* and *XPREP* (Bruker, 2004 ▸); program(s) used to solve structure: *SHELXS97* (Sheldrick, 2008 ▸); program(s) used to refine structure: *SHELXL2014* (Sheldrick, 2015 ▸); molecular graphics: *ORTEP-3 for Windows* (Farrugia, 2012 ▸); software used to prepare material for publication: *PLATON* (Spek, 2009 ▸).

## Supplementary Material

Crystal structure: contains datablock(s) I, 2R. DOI: 10.1107/S2056989015024172/bg2572sup1.cif

Structure factors: contains datablock(s) I. DOI: 10.1107/S2056989015024172/bg2572Isup2.hkl

Supporting information file. DOI: 10.1107/S2056989015024172/bg2572Isup3.cml

**Fig. 1.**
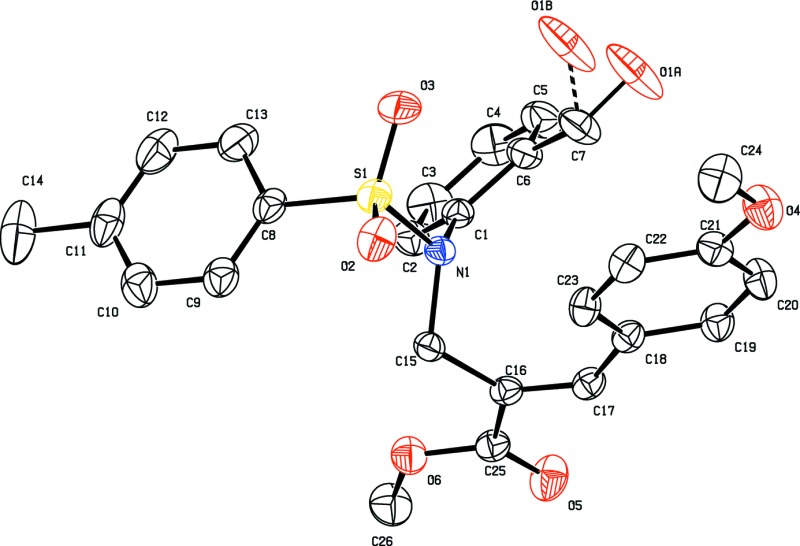
The molecular structure of the title compound showing 30% probability displacement ellipsoids for non-H atoms

. DOI: 10.1107/S2056989015024172/bg2572fig1.tif
The mol­ecular structure of the title compound showing 30% probability displacement ellipsoids for non-H atoms

**Fig. 2.**
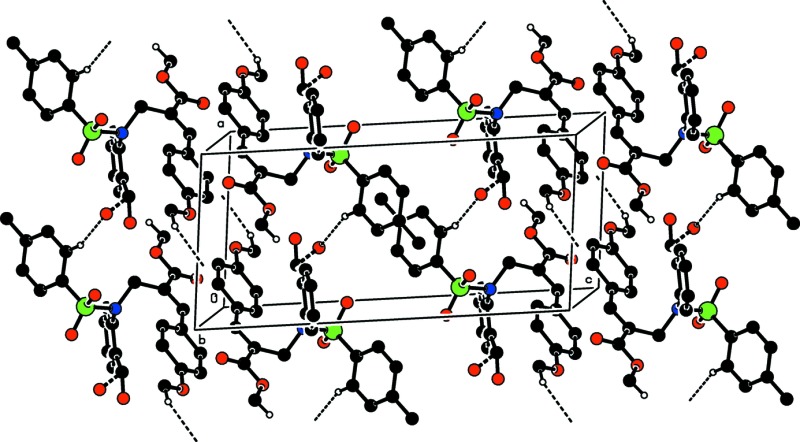
Crystal packing diagram. H atoms not involved in hydrogen bonding (dashed lines) have been omitted for clarity.

. DOI: 10.1107/S2056989015024172/bg2572fig2.tif
Crystal packing diagram. H atoms not involved in hydrogen bonding (dashed lines) have been omitted for clarity.

CCDC reference: 1442750

Additional supporting information:  crystallographic information; 3D view; checkCIF report

## Figures and Tables

**Table 1:** Hydrogen-bond geometry (Å, °)

*D*—H⋯*A*	*D*—H	H⋯*A*	*D*⋯*A*	*D*—H⋯*A*
C9—H9⋯O1*B* ^i^	0.93	2.50	3.397 (7)	162
C15—H15*A*⋯O6	0.97	2.24	2.7322 (19)	111
C24—H24*A*⋯O4^ii^	0.96	2.52	3.341 (3)	143

Symmetry codes: (i) 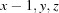; (ii) 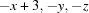.

## supplementary crystallographic information

### S1. Comment

Sulfonamide drugs are widely used for the treatment of certain infections caused by Gram-positive and Gram-negative micro-organisms, some fungi, and certain protozoa (Korolkovas *et al.*, 1988, Mandell & Sande 1992). One of the Sulfonamide derivatives (epoxysulphonamides and ethynesulphonamides) shows anti-filarial activity (Radembino *et al.*, 1997). In view of this biological importance, the crystal structure of the title compound has been determined and the results are presented here. The molecular structure of the title compound is shown in Fig. 1.The S1 atom shows a distorted tetrahedral geometry, with O2—S1—O3 [119.4 (1)°] and N1—S1—C8 [107.1 (1)°] angles deviating from ideal tetrahedral values are attributed to the Thrope-Ingold effect (Bassindale *et al.*, 1984). The sum of bond angles around N1 (348.3°) indicates that N1 is in sp2 hybridization. The sulfonyl bound phenyl (C8–C13) ring forms dihedral angles of 41.1 (1)° and 83.3 (1)°, respectively, with the formyl phenyl (C1–C6) and phenyl (C18—C23) rings. The dihedral angle between formyl phenyl and phenyl rings is 47.6 (1)°. The geometric parameters agree well with those reported for similar structures (Ranjith *et al.*, 2009; Madhanraj *et al.*, 2011). Crystal packing is stabilized by C19—H19···O5 and C24—H24A···O4 inter molecular hydrogen bond interaction. (Shown in Fig.2).

### S2. Experimental

A solution of *N*-(formylphenyl)(4-methylbenzene)sulfonamide (1 mmol, 0.275 g) and potassium carbonate (1.5 mmol, 0.207 g) in acetonitrile solvent was stirred for 15 min at room temperature. To this solution, methyl(2*Z*)-2-(bromomethyl)-3-(4-methoxyphenyl)prop-2-enoate (1.2 mmol, 0.342 g) was added dropwise till the addition was complete. After the completion of the reaction, as indicated by TLC, acetonitrile was evaporated. EtOAc (15 ml) and water (15 ml) were added to the crude mass. The organic layer was dried over anhydrous sodium sulfate. Removal of solvent led to the crude product, which was purified through pad of silica gel (100–200mesh) using ethylacetate and hexane(1:9) as solvents. The pure title compound was obtained as a colourless solid (0.426 g, 89% yield). Recrystallization was carried out using ethylacetate as solvent.

### S3. Refinement

All H atoms were fixed and refined using a riding model with C—H ranging from 0.93 to 0.97 Å. The formylphenyl O1 (O1A, O1B) and H7 (H7A, H7B) atoms appear disordered over two sites with s.o.f 0.740 (4) and 0.260 (4), respectively. O1A and O1B were refined with restraints in their anisotropic thermal parameters and C-O distances.

### Crystal data

**Table:**

C_26_H_25_NO_6_S	*Z* = 2
*M**_r_* = 479.53	*F*(000) = 504
Triclinic, *P*1	*D*_x_ = 1.307 Mg m^−^^3^
Hall symbol: -P 1	Mo *K*α radiation, λ = 0.71073 Å
*a* = 8.3501 (2) Å	Cell parameters from 8834 reflections
*b* = 8.4859 (2) Å	θ = 2.6–31.2°
*c* = 17.6814 (4) Å	µ = 0.17 mm^−^^1^
α = 84.424 (1)°	*T* = 293 K
β = 80.952 (1)°	Block, colourless
γ = 80.954 (1)°	0.25 × 0.20 × 0.20 mm
*V* = 1218.52 (5) Å^3^	

### Data collection

**Table:**

Bruker Kappa APEXII CCD diffractometer	5519 independent reflections
Radiation source: fine-focus sealed tube	4232 reflections with *I* > 2σ(*I*)
Graphite monochromator	*R*_int_ = 0.028
ω and φ scan	θ_max_ = 27.5°, θ_min_ = 2.3°
Absorption correction: multi-scan (*SADABS*; Bruker 2004)	*h* = −10→10
*T*_min_ = 0.979, *T*_max_ = 0.983	*k* = −11→11
23816 measured reflections	*l* = −22→22

### Refinement

**Table:**

Refinement on *F*^2^	13 restraints
Least-squares matrix: full	Hydrogen site location: mixed
*R*[*F*^2^ > 2σ(*F*^2^)] = 0.044	H-atom parameters constrained
*wR*(*F*^2^) = 0.135	*w* = 1/[σ^2^(*F*_o_^2^) + (0.0713*P*)^2^ + 0.275*P*] where *P* = (*F*_o_^2^ + 2*F*_c_^2^)/3
*S* = 1.02	(Δ/σ)_max_ < 0.001
5519 reflections	Δρ_max_ = 0.34 e Å^−^^3^
314 parameters	Δρ_min_ = −0.36 e Å^−^^3^

### Special details

**Table:**

Geometry. All esds (except the esd in the dihedral angle between two l.s. planes) are estimated using the full covariance matrix. The cell esds are taken into account individually in the estimation of esds in distances, angles and torsion angles; correlations between esds in cell parameters are only used when they are defined by crystal symmetry. An approximate (isotropic) treatment of cell esds is used for estimating esds involving l.s. planes.
Refinement. Refinement of *F*^2^ against ALL reflections. The weighted *R*-factor *wR* and goodness of fit *S* are based on *F*^2^, conventional *R*-factors *R* are based on *F*, with *F* set to zero for negative *F*^2^. The threshold expression of *F*^2^ > σ(*F*^2^) is used only for calculating *R*-factors(gt) *etc*. and is not relevant to the choice of reflections for refinement. *R*-factors based on *F*^2^ are statistically about twice as large as those based on *F*, and *R*- factors based on ALL data will be even larger.

### Fractional atomic coordinates and isotropic or equivalent isotropic displacement parameters (Å^2^)

**Table:**

	*x*	*y*	*z*	*U*_iso_*/*U*_eq_	Occ. (<1)
S1	0.89631 (5)	0.51292 (5)	0.33325 (2)	0.04835 (15)	
O1A	1.4384 (2)	0.5552 (4)	0.2246 (2)	0.1151 (13)	0.740 (4)
H7A	1.2277	0.5012	0.2267	0.080*	0.740 (4)
O1B	1.3729 (9)	0.4953 (8)	0.2764 (4)	0.1151 (13)	0.260 (4)
H7B	1.2365	0.5460	0.1946	0.080*	0.260 (4)
O2	0.82045 (18)	0.40111 (14)	0.30104 (8)	0.0609 (4)	
O3	1.04550 (18)	0.46004 (16)	0.36347 (8)	0.0696 (4)	
O4	1.35510 (18)	0.15273 (16)	0.03718 (9)	0.0700 (4)	
O5	0.83811 (19)	1.06484 (15)	0.08082 (8)	0.0693 (4)	
O6	0.66997 (15)	1.02738 (14)	0.18898 (7)	0.0565 (3)	
N1	0.93431 (15)	0.65272 (14)	0.26408 (7)	0.0379 (3)	
C1	1.03147 (19)	0.76893 (18)	0.27928 (9)	0.0396 (3)	
C2	0.9569 (2)	0.9142 (2)	0.30551 (11)	0.0525 (4)	
H2	0.8432	0.9362	0.3149	0.063*	
C3	1.0505 (3)	1.0272 (2)	0.31794 (13)	0.0677 (6)	
H3	0.9996	1.1253	0.3352	0.081*	
C4	1.2173 (3)	0.9952 (3)	0.30501 (14)	0.0720 (6)	
H4	1.2801	1.0710	0.3139	0.086*	
C5	1.2920 (2)	0.8516 (3)	0.27898 (12)	0.0651 (5)	
H5	1.4059	0.8307	0.2703	0.078*	
C6	1.2016 (2)	0.7363 (2)	0.26528 (10)	0.0494 (4)	
C7	1.2889 (2)	0.5845 (3)	0.23465 (13)	0.0674 (6)	
C8	0.7539 (2)	0.6044 (2)	0.40537 (9)	0.0493 (4)	
C9	0.5901 (2)	0.6237 (2)	0.39958 (11)	0.0587 (5)	
H9	0.5535	0.5855	0.3588	0.070*	
C10	0.4797 (3)	0.7018 (3)	0.45622 (13)	0.0703 (6)	
H10	0.3682	0.7165	0.4528	0.084*	
C11	0.5324 (3)	0.7579 (2)	0.51727 (12)	0.0702 (6)	
C12	0.6966 (4)	0.7339 (3)	0.52188 (13)	0.0793 (7)	
H12	0.7333	0.7691	0.5634	0.095*	
C13	0.8076 (3)	0.6594 (3)	0.46667 (11)	0.0678 (6)	
H13	0.9190	0.6457	0.4703	0.081*	
C14	0.4101 (4)	0.8423 (3)	0.57835 (16)	0.1047 (10)	
H14A	0.3053	0.8683	0.5608	0.157*	
H14B	0.4473	0.9388	0.5882	0.157*	
H14C	0.4002	0.7734	0.6247	0.157*	
C15	0.79663 (18)	0.71501 (18)	0.22006 (9)	0.0401 (3)	
H15A	0.7147	0.7856	0.2509	0.048*	
H15B	0.7455	0.6265	0.2089	0.048*	
C16	0.85673 (19)	0.80473 (18)	0.14613 (9)	0.0392 (3)	
C17	0.9703 (2)	0.74544 (19)	0.08975 (9)	0.0430 (4)	
H17	0.9941	0.8196	0.0488	0.052*	
C18	1.0633 (2)	0.58688 (19)	0.08059 (9)	0.0424 (4)	
C19	1.2034 (2)	0.5730 (2)	0.02594 (10)	0.0524 (4)	
H19	1.2334	0.6645	−0.0027	0.063*	
C20	1.2982 (2)	0.4287 (2)	0.01316 (12)	0.0587 (5)	
H20	1.3920	0.4232	−0.0232	0.070*	
C21	1.2547 (2)	0.2907 (2)	0.05425 (11)	0.0507 (4)	
C22	1.1149 (2)	0.2998 (2)	0.10759 (10)	0.0521 (4)	
H22	1.0840	0.2076	0.1351	0.062*	
C23	1.0211 (2)	0.4462 (2)	0.12001 (10)	0.0489 (4)	
H23	0.9265	0.4510	0.1559	0.059*	
C24	1.3116 (3)	0.0075 (2)	0.07567 (14)	0.0790 (7)	
H24A	1.3916	−0.0803	0.0580	0.119*	
H24B	1.2059	−0.0071	0.0650	0.119*	
H24C	1.3080	0.0117	0.1300	0.119*	
C25	0.7907 (2)	0.97716 (19)	0.13359 (9)	0.0445 (4)	
C26	0.6048 (3)	1.1955 (2)	0.18269 (14)	0.0693 (6)	
H26A	0.5197	1.2190	0.2250	0.104*	
H26B	0.5607	1.2211	0.1353	0.104*	
H26C	0.6908	1.2579	0.1836	0.104*	

### Atomic displacement parameters (Å^2^)

**Table:**

	*U*^11^	*U*^22^	*U*^33^	*U*^12^	*U*^13^	*U*^23^
S1	0.0578 (3)	0.0350 (2)	0.0469 (2)	0.00262 (17)	−0.00629 (19)	0.00590 (16)
O1A	0.0336 (12)	0.118 (2)	0.199 (3)	0.0111 (12)	−0.0125 (15)	−0.082 (2)
O1B	0.0336 (12)	0.118 (2)	0.199 (3)	0.0111 (12)	−0.0125 (15)	−0.082 (2)
O2	0.0854 (10)	0.0349 (6)	0.0604 (8)	−0.0140 (6)	−0.0004 (7)	−0.0018 (5)
O3	0.0716 (9)	0.0610 (8)	0.0674 (8)	0.0168 (7)	−0.0208 (7)	0.0137 (7)
O4	0.0663 (9)	0.0500 (7)	0.0866 (10)	0.0013 (6)	0.0017 (8)	−0.0059 (7)
O5	0.0902 (10)	0.0450 (7)	0.0628 (8)	−0.0074 (7)	0.0068 (7)	0.0127 (6)
O6	0.0576 (8)	0.0441 (6)	0.0602 (8)	0.0031 (5)	−0.0009 (6)	0.0047 (6)
N1	0.0368 (7)	0.0346 (6)	0.0406 (7)	−0.0006 (5)	−0.0064 (5)	0.0006 (5)
C1	0.0417 (8)	0.0379 (7)	0.0383 (8)	−0.0002 (6)	−0.0089 (6)	−0.0023 (6)
C2	0.0524 (10)	0.0443 (9)	0.0589 (11)	0.0044 (7)	−0.0095 (8)	−0.0106 (8)
C3	0.0856 (16)	0.0428 (10)	0.0773 (14)	−0.0027 (9)	−0.0198 (12)	−0.0158 (9)
C4	0.0803 (16)	0.0650 (13)	0.0815 (15)	−0.0261 (11)	−0.0252 (12)	−0.0128 (11)
C5	0.0493 (11)	0.0781 (14)	0.0737 (13)	−0.0138 (10)	−0.0171 (10)	−0.0125 (11)
C6	0.0417 (9)	0.0550 (10)	0.0522 (10)	0.0012 (7)	−0.0134 (8)	−0.0104 (8)
C7	0.0425 (10)	0.0754 (13)	0.0857 (15)	0.0117 (9)	−0.0194 (10)	−0.0296 (11)
C8	0.0617 (11)	0.0423 (8)	0.0395 (9)	−0.0038 (7)	−0.0039 (8)	0.0074 (7)
C9	0.0653 (12)	0.0546 (10)	0.0525 (10)	−0.0110 (9)	−0.0007 (9)	0.0058 (8)
C10	0.0643 (13)	0.0620 (12)	0.0742 (14)	−0.0079 (10)	0.0090 (11)	0.0136 (10)
C11	0.0995 (18)	0.0490 (10)	0.0504 (11)	−0.0066 (11)	0.0152 (11)	0.0061 (9)
C12	0.111 (2)	0.0733 (14)	0.0500 (12)	−0.0041 (13)	−0.0071 (12)	−0.0084 (10)
C13	0.0805 (15)	0.0700 (13)	0.0512 (11)	−0.0029 (11)	−0.0138 (10)	−0.0026 (10)
C14	0.134 (2)	0.0742 (16)	0.0836 (17)	−0.0069 (16)	0.0461 (17)	−0.0058 (14)
C15	0.0339 (8)	0.0409 (8)	0.0436 (8)	−0.0044 (6)	−0.0060 (6)	0.0047 (6)
C16	0.0403 (8)	0.0395 (8)	0.0402 (8)	−0.0108 (6)	−0.0108 (7)	0.0022 (6)
C17	0.0499 (9)	0.0417 (8)	0.0394 (8)	−0.0138 (7)	−0.0086 (7)	0.0019 (6)
C18	0.0479 (9)	0.0440 (8)	0.0383 (8)	−0.0126 (7)	−0.0085 (7)	−0.0042 (6)
C19	0.0540 (10)	0.0483 (9)	0.0529 (10)	−0.0127 (8)	0.0003 (8)	0.0023 (8)
C20	0.0481 (10)	0.0584 (11)	0.0652 (12)	−0.0090 (8)	0.0068 (9)	−0.0041 (9)
C21	0.0499 (10)	0.0464 (9)	0.0566 (10)	−0.0042 (7)	−0.0105 (8)	−0.0086 (8)
C22	0.0659 (12)	0.0416 (8)	0.0501 (10)	−0.0156 (8)	−0.0054 (8)	−0.0033 (7)
C23	0.0554 (10)	0.0464 (9)	0.0452 (9)	−0.0158 (7)	0.0026 (8)	−0.0075 (7)
C24	0.0998 (18)	0.0466 (11)	0.0829 (15)	−0.0004 (11)	0.0001 (13)	−0.0043 (10)
C25	0.0486 (9)	0.0414 (8)	0.0442 (9)	−0.0079 (7)	−0.0102 (7)	0.0013 (7)
C26	0.0743 (14)	0.0447 (10)	0.0818 (14)	0.0054 (9)	−0.0061 (11)	−0.0011 (9)

### Geometric parameters (Å, º)

**Table:**

S1—O3	1.4233 (14)	C11—C12	1.368 (3)
S1—O2	1.4259 (14)	C11—C14	1.515 (3)
S1—N1	1.6486 (13)	C12—C13	1.365 (3)
S1—C8	1.7517 (18)	C12—H12	0.9300
O1A—C7	1.222 (3)	C13—H13	0.9300
O1B—C7	1.223 (3)	C14—H14A	0.9600
O4—C21	1.359 (2)	C14—H14B	0.9600
O4—C24	1.419 (3)	C14—H14C	0.9600
O5—C25	1.193 (2)	C15—C16	1.504 (2)
O6—C25	1.339 (2)	C15—H15A	0.9700
O6—C26	1.445 (2)	C15—H15B	0.9700
N1—C1	1.440 (2)	C16—C17	1.340 (2)
N1—C15	1.4891 (18)	C16—C25	1.489 (2)
C1—C2	1.379 (2)	C17—C18	1.454 (2)
C1—C6	1.391 (2)	C17—H17	0.9300
C2—C3	1.382 (3)	C18—C23	1.389 (2)
C2—H2	0.9300	C18—C19	1.392 (2)
C3—C4	1.363 (3)	C19—C20	1.368 (3)
C3—H3	0.9300	C19—H19	0.9300
C4—C5	1.366 (3)	C20—C21	1.384 (3)
C4—H4	0.9300	C20—H20	0.9300
C5—C6	1.386 (3)	C21—C22	1.377 (3)
C5—H5	0.9300	C22—C23	1.378 (2)
C6—C7	1.483 (3)	C22—H22	0.9300
C7—H7A	0.9672	C23—H23	0.9300
C7—H7B	0.9933	C24—H24A	0.9600
C8—C9	1.371 (3)	C24—H24B	0.9600
C8—C13	1.383 (3)	C24—H24C	0.9600
C9—C10	1.392 (3)	C26—H26A	0.9600
C9—H9	0.9300	C26—H26B	0.9600
C10—C11	1.378 (3)	C26—H26C	0.9600
C10—H10	0.9300		
			
O3—S1—O2	119.46 (9)	C8—C13—H13	120.1
O3—S1—N1	106.81 (8)	C11—C14—H14A	109.5
O2—S1—N1	106.18 (7)	C11—C14—H14B	109.5
O3—S1—C8	108.18 (9)	H14A—C14—H14B	109.5
O2—S1—C8	108.44 (9)	C11—C14—H14C	109.5
N1—S1—C8	107.16 (7)	H14A—C14—H14C	109.5
C21—O4—C24	117.95 (16)	H14B—C14—H14C	109.5
C25—O6—C26	116.06 (14)	N1—C15—C16	110.86 (12)
C1—N1—C15	116.00 (11)	N1—C15—H15A	109.5
C1—N1—S1	116.79 (10)	C16—C15—H15A	109.5
C15—N1—S1	115.48 (10)	N1—C15—H15B	109.5
C2—C1—C6	119.77 (16)	C16—C15—H15B	109.5
C2—C1—N1	120.41 (14)	H15A—C15—H15B	108.1
C6—C1—N1	119.79 (14)	C17—C16—C25	115.34 (14)
C1—C2—C3	120.25 (18)	C17—C16—C15	126.06 (14)
C1—C2—H2	119.9	C25—C16—C15	118.56 (14)
C3—C2—H2	119.9	C16—C17—C18	132.06 (14)
C4—C3—C2	120.22 (18)	C16—C17—H17	114.0
C4—C3—H3	119.9	C18—C17—H17	114.0
C2—C3—H3	119.9	C23—C18—C19	116.85 (15)
C3—C4—C5	119.86 (19)	C23—C18—C17	125.39 (15)
C3—C4—H4	120.1	C19—C18—C17	117.72 (15)
C5—C4—H4	120.1	C20—C19—C18	121.79 (16)
C4—C5—C6	121.33 (19)	C20—C19—H19	119.1
C4—C5—H5	119.3	C18—C19—H19	119.1
C6—C5—H5	119.3	C19—C20—C21	120.10 (17)
C5—C6—C1	118.57 (16)	C19—C20—H20	119.9
C5—C6—C7	119.11 (17)	C21—C20—H20	119.9
C1—C6—C7	122.31 (16)	O4—C21—C22	124.42 (17)
O1A—C7—C6	121.8 (2)	O4—C21—C20	115.99 (17)
O1B—C7—C6	117.2 (4)	C22—C21—C20	119.57 (16)
O1A—C7—H7A	118.0	C21—C22—C23	119.62 (16)
C6—C7—H7A	120.0	C21—C22—H22	120.2
O1B—C7—H7B	123.3	C23—C22—H22	120.2
C6—C7—H7B	114.0	C22—C23—C18	122.03 (16)
C9—C8—C13	120.62 (18)	C22—C23—H23	119.0
C9—C8—S1	119.52 (15)	C18—C23—H23	119.0
C13—C8—S1	119.85 (16)	O4—C24—H24A	109.5
C8—C9—C10	118.4 (2)	O4—C24—H24B	109.5
C8—C9—H9	120.8	H24A—C24—H24B	109.5
C10—C9—H9	120.8	O4—C24—H24C	109.5
C11—C10—C9	121.3 (2)	H24A—C24—H24C	109.5
C11—C10—H10	119.4	H24B—C24—H24C	109.5
C9—C10—H10	119.4	O5—C25—O6	122.00 (15)
C12—C11—C10	118.6 (2)	O5—C25—C16	125.15 (16)
C12—C11—C14	121.0 (2)	O6—C25—C16	112.85 (13)
C10—C11—C14	120.4 (3)	O6—C26—H26A	109.5
C13—C12—C11	121.3 (2)	O6—C26—H26B	109.5
C13—C12—H12	119.4	H26A—C26—H26B	109.5
C11—C12—H12	119.4	O6—C26—H26C	109.5
C12—C13—C8	119.7 (2)	H26A—C26—H26C	109.5
C12—C13—H13	120.1	H26B—C26—H26C	109.5
			
O3—S1—N1—C1	−43.28 (13)	C9—C10—C11—C12	−0.7 (3)
O2—S1—N1—C1	−171.79 (11)	C9—C10—C11—C14	−179.89 (19)
C8—S1—N1—C1	72.47 (13)	C10—C11—C12—C13	1.5 (3)
O3—S1—N1—C15	175.04 (11)	C14—C11—C12—C13	−179.3 (2)
O2—S1—N1—C15	46.53 (13)	C11—C12—C13—C8	−1.1 (3)
C8—S1—N1—C15	−69.21 (13)	C9—C8—C13—C12	−0.1 (3)
C15—N1—C1—C2	45.8 (2)	S1—C8—C13—C12	178.51 (16)
S1—N1—C1—C2	−95.71 (16)	C1—N1—C15—C16	54.01 (17)
C15—N1—C1—C6	−132.35 (15)	S1—N1—C15—C16	−164.00 (11)
S1—N1—C1—C6	86.17 (16)	N1—C15—C16—C17	58.9 (2)
C6—C1—C2—C3	0.0 (3)	N1—C15—C16—C25	−118.85 (15)
N1—C1—C2—C3	−178.15 (16)	C25—C16—C17—C18	179.51 (16)
C1—C2—C3—C4	−0.6 (3)	C15—C16—C17—C18	1.7 (3)
C2—C3—C4—C5	0.6 (4)	C16—C17—C18—C23	19.7 (3)
C3—C4—C5—C6	0.0 (4)	C16—C17—C18—C19	−162.71 (17)
C4—C5—C6—C1	−0.6 (3)	C23—C18—C19—C20	−2.0 (3)
C4—C5—C6—C7	178.0 (2)	C17—C18—C19—C20	−179.77 (17)
C2—C1—C6—C5	0.6 (3)	C18—C19—C20—C21	0.9 (3)
N1—C1—C6—C5	178.73 (16)	C24—O4—C21—C22	1.3 (3)
C2—C1—C6—C7	−177.97 (18)	C24—O4—C21—C20	−177.5 (2)
N1—C1—C6—C7	0.2 (3)	C19—C20—C21—O4	179.36 (18)
C5—C6—C7—O1A	3.6 (4)	C19—C20—C21—C22	0.5 (3)
C1—C6—C7—O1A	−177.9 (3)	O4—C21—C22—C23	−179.51 (17)
C5—C6—C7—O1B	66.3 (5)	C20—C21—C22—C23	−0.7 (3)
C1—C6—C7—O1B	−115.1 (5)	C21—C22—C23—C18	−0.4 (3)
O3—S1—C8—C9	−161.09 (14)	C19—C18—C23—C22	1.7 (3)
O2—S1—C8—C9	−30.16 (15)	C17—C18—C23—C22	179.32 (16)
N1—S1—C8—C9	84.07 (15)	C26—O6—C25—O5	−2.3 (3)
O3—S1—C8—C13	20.28 (17)	C26—O6—C25—C16	177.19 (15)
O2—S1—C8—C13	151.21 (15)	C17—C16—C25—O5	−4.8 (3)
N1—S1—C8—C13	−94.56 (15)	C15—C16—C25—O5	173.22 (17)
C13—C8—C9—C10	0.9 (3)	C17—C16—C25—O6	175.70 (14)
S1—C8—C9—C10	−177.74 (13)	C15—C16—C25—O6	−6.3 (2)
C8—C9—C10—C11	−0.5 (3)		

### Hydrogen-bond geometry (Å, º)

**Table:**

*D*—H···*A*	*D*—H	H···*A*	*D*···*A*	*D*—H···*A*
C9—H9···O1*B*^i^	0.93	2.50	3.397 (7)	162
C15—H15*A*···O6	0.97	2.24	2.7322 (19)	111
C24—H24*A*···O4^ii^	0.96	2.52	3.341 (3)	143

Symmetry codes: (i) *x*−1, *y*, *z*; (ii) −*x*+3, −*y*, −*z*.
