# Supplementary figures and images for: Crystal structure of methyl (2Z)-2-{[N-(2-formyl­phen­yl)-4-methyl­benzene­sulfonamido]­meth­yl}-3-(4-meth­oxy­phen­yl)prop-2-enoate
Source: Acta Crystallogr E Crystallogr Commun. 2015 Dec 31;71(Pt 12):o1088–9. doi: 10.1107/S2056989015024172 (PMC4719993; doi:10.1107/S2056989015024172)

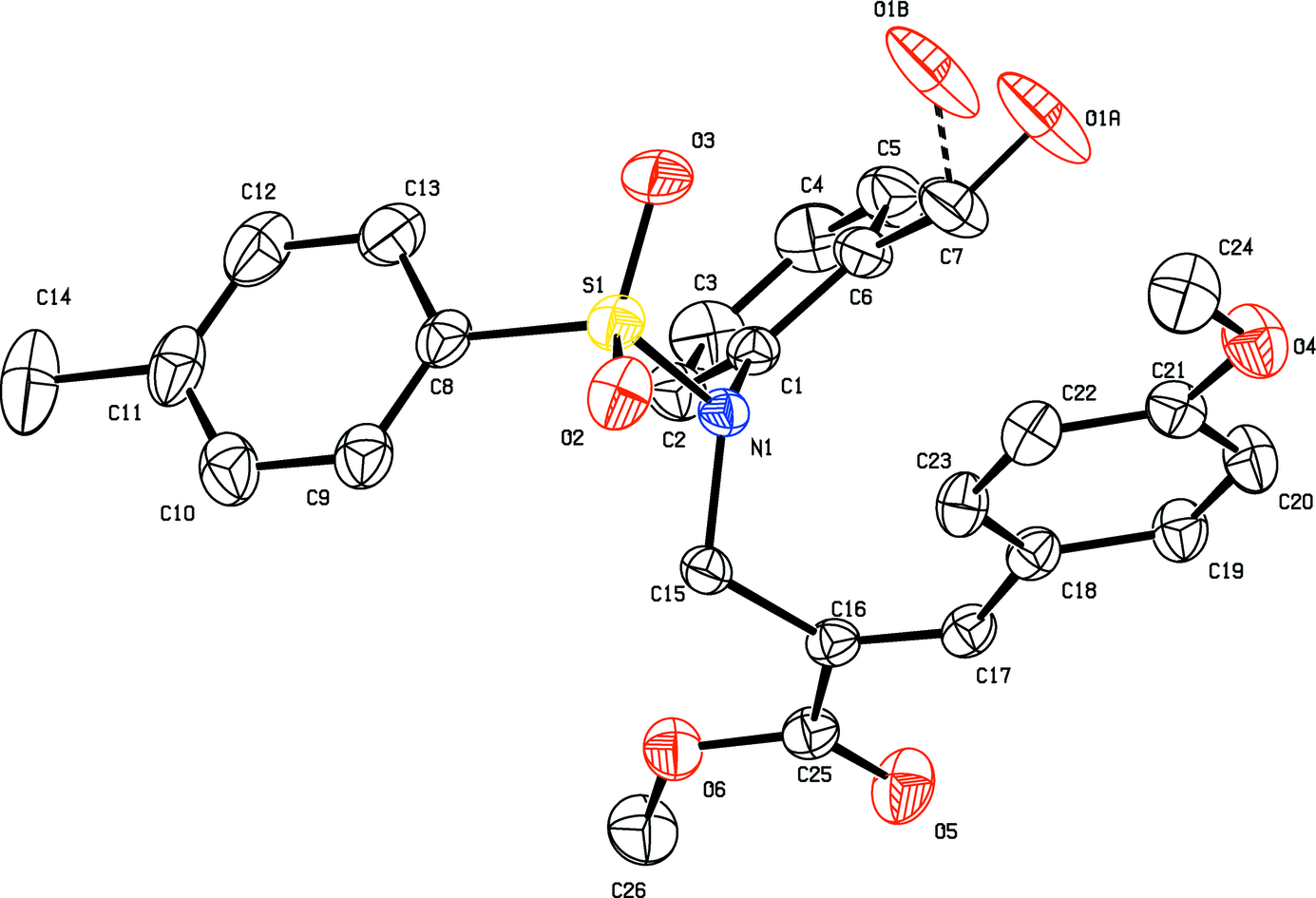

Supplement: Supplementary file 4 [file e-71-o1088-fig1.tif]

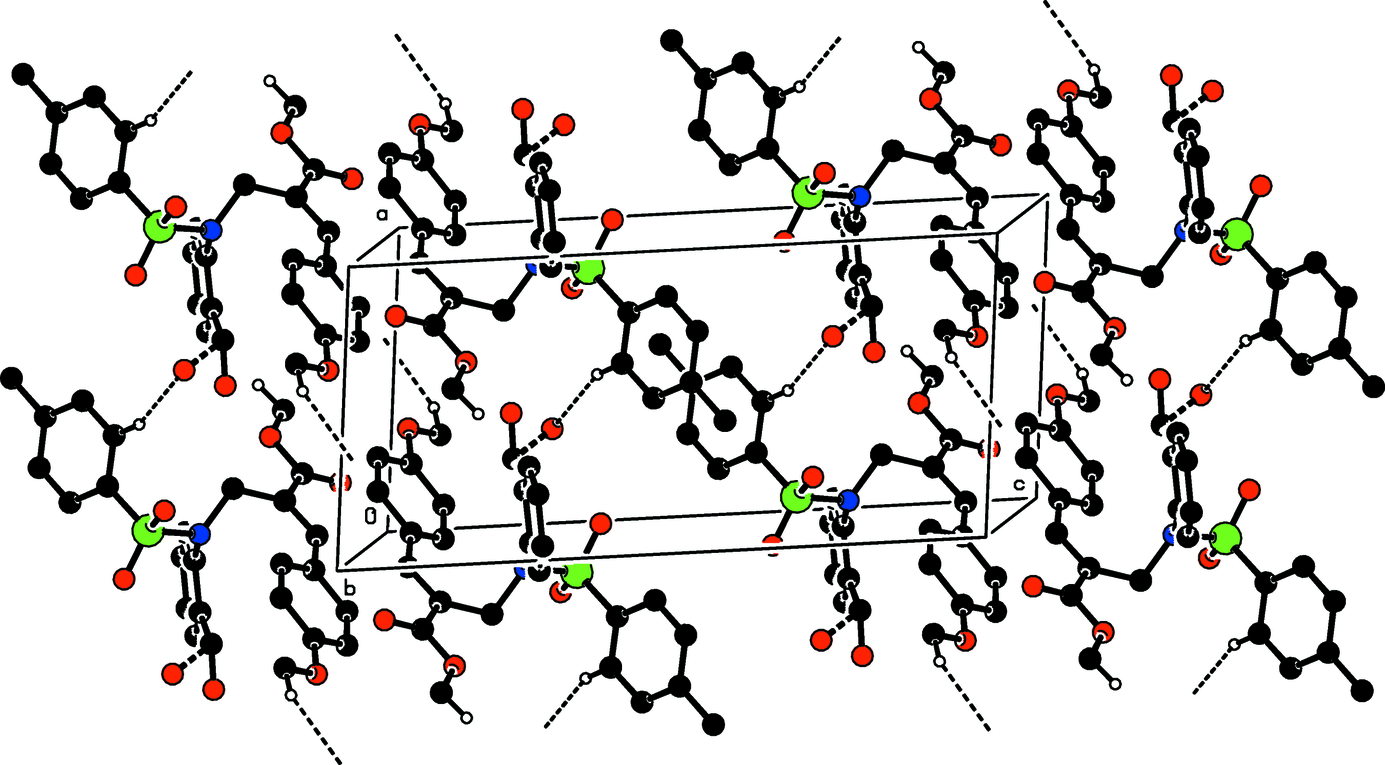

Supplement: Supplementary file 5 [file e-71-o1088-fig2.tif]
